# Role of Non-invasive Ventilation (NIV) in Managing Acute Exacerbations of Chronic Obstructive Pulmonary Disease (COPD): A Systematic Review

**DOI:** 10.7759/cureus.67418

**Published:** 2024-08-21

**Authors:** Shahad Abduljalil Abualhamael, Ahmed T Alasmi, Abdulrahman F Alqurayqiri, Abdulillah A Alzahrani, Ahmed D Alsehli, Abdulaziz H Althikra, Safwan O Alsadi, Mussab Z Almaghrabi, Turki S Alhamdi, Meshal D Alsehli

**Affiliations:** 1 Department of Internal Medicine, Faculty of Medicine in Rabigh, King Abdulaziz University, Jeddah, SAU; 2 Department of Medicine, Faculty of Medicine in Rabigh, King Abdulaziz University, Jeddah, SAU

**Keywords:** high-flow nasal therapy, respiratory failure, niv (non-invasive ventilation), chronic obstructive pulmonary disease, copd

## Abstract

Chronic obstructive pulmonary disease (COPD) is a significant global health issue that is characterized by airflow constriction and breathing difficulties. Non-invasive ventilation (NIV) is a recommended treatment for acute exacerbations of COPD (AECOPD), offering benefits over invasive mechanical ventilation (IMV). We aimed to evaluate the effectiveness, safety, and impact of NIV in managing AECOPD. The study adheres to the Preferred Reporting Items for Systematic Reviews and Meta-Analyses (PRISMA) guidelines. We searched PubMed, Medline, Cochrane Library, Embase, and Google Scholar for relevant studies published between 2015 and 2024. Inclusion criteria focused on studies involving AECOPD patients treated with NIV, including randomized controlled, cohort, and observational studies. We included 10 studies that fit our inclusion criteria for a thorough review. From the studies selected, NIV demonstrated significant reductions in mortality rates, intubation rates, and hospital stays compared to IMV. Albeit the need to train healthcare providers is essential, high adherence to NIV guidelines was observed. Different NIV modes showed comparable efficacy, and structured weaning protocols reduced relapse rates. NIV is a highly effective and safe treatment for patients with AECOPD than IMV. High-flow nasal therapy (HFNT) is a viable alternative for patients intolerant to NIV. Further research should standardize treatment protocols and optimize NIV use in clinical practice.

## Introduction and background

Chronic obstructive pulmonary disease (COPD) is a progressive, highly prevalent alveolus condition characterised by restricted airflow and breathing. It is often called emphysema or chronic bronchitis in the USA [1]. COPD is an important public health issue globally. The disease affects millions of people and is highly prevalent in low- and middle-income countries. It is the third leading cause of death worldwide, accounting for 3.23 million deaths in 2019. COPD disproportionately affects individuals under 70 years of age, with close to 90% of COPD-related deaths occurring in this demographic [2]. It is also the seventh leading cause of disability worldwide.

The primary causes are smoking and air pollution, with tobacco smoking accounting for every 7 out of 10 COPD cases in high-income countries and about 35% in low-and-middle-income regions [3]. COPD symptoms include chronic cough, difficulty breathing, wheezing, and fatigue. COPD manifests as chronic bronchitis and emphysema, but diagnosing it can be challenging due to symptom overlap with asthma and other lung diseases [4]. While incurable, COPD is manageable with medications, non-invasive ventilation (NIV), and lifestyle changes. The most effective way to prevent COPD, however, is to avoid or stop smoking tobacco [4].

NIV is a widely endorsed, evidence-based treatment for acute respiratory failure (ARF) due to acute exacerbation of chronic obstructive pulmonary disease (AECOPD) [5]. The method is quite effective in patients with acute hypercapnic respiratory failure (AHRF) by improving the exchange of gases, minimising the need to keep breathing, and effectively reducing mortality and hospitalisation [6]. Medical evidence indicates that NIV is more effective than invasive ventilation because it has fewer complications [7,8]. NIV is guideline-recommended because of its evidence-backed effectiveness [9] when treating patients with ARF because of aggravated COPD. The guideline committee developed the GRADE methodology that prescribed using NIV in patients presenting with ARF. It supports its implementation in various clinical scenarios, including immunocompromised patients, exacerbated COPD, and post-operative care [9].

Studies show that NIV treatment for patients with AECOPD significantly decreases mortality rates, shortens hospital stays, and has a lower risk of complications than IMV [9]. This method has become a cornerstone in treating severe COPD exacerbations and reducing healthcare costs.

However, the actual effectiveness of NIV on patients with ARF secondary to AECOPD is still uncertain. Studies conducted on the use of NIV vis-à-vis invasive ventilation and other methods have yielded mixed results. This systematic review, therefore, aims to assess the role of NIV in managing acute exacerbations of COPD, evaluating its effectiveness, safety, and impact on patient outcomes.

## Review

Materials and Methods

Literature Search Strategy

This study utilised the guidelines set forth by Preferred Reporting Items for Systematic Reviews and Meta-Analyses (PRISMA), through which five medical databases were searched for relevant scholarly publications published between 2015 and 2024. These databases included PubMed, Medline, Cochrane Library, Embase, and Google Scholar. A combination of keywords was used in searching for relevant articles for review; general terms included: “COPD” / ”Chronic Obstructive Pulmonary Disease” / “acute exacerbations” / “respiratory failure”. Intervention terms: “Non-Invasive Ventilation” / “NIV” / “NIVvsIMV” / “mechanical ventilation”. Outcome-specific terms: “mortality rates” / “intubation rates” / “hospital stay duration” / “complications” / “patient outcomes”.

Eligibility, Data Extraction, and Management

The retrieved articles were rigorously assessed for eligibility by comparing them with the pre-defined inclusion and exclusion criteria that were agreed upon by all the researchers involved in this study.

Inclusion criteria: Studies on patients diagnosed with AECOPD that investigate the use of NIV as a treatment were eligible. Eligible studies included randomised controlled trials, cohort, observational, retrospective, and observational studies. The primary outcomes of interest are mortality rates, intubation rates, length of hospital stay, and complication incidences. Studies must compare NIV with standard care or other interventions. Only articles published in English from 2015 onwards were considered.

Exclusion criteria: Studies that did not focus on patients with acute exacerbations of COPD or if they did not investigate the use of NIV as a treatment were excluded. Studies were excluded if they were case reports, non-peered review articles, systematic reviews, abstracts, conference papers, and editorials. Research articles that did not compare NIV with standard care or other interventions or did not have outcomes such as mortality rates, intubation rates, length of hospital stay, and incidence of complications reported on prima were also excluded. Non-English language publications and studies published before 2015 were not considered. Studies involving pediatric patients or those with conditions other than COPD were excluded to ensure relevance and specificity to the review objectives.

The above eligibility criteria were developed to ensure that only high-quality, relevant and recent studies offered meaningful information on the role of NIV in managing patients with AECOPD. All the PRISMA guidelines were observed and strictly followed. Disputes were resolved promptly through inclusive discussions upon which a consensus was reached. The researchers retrieved and documented all the data relevant to this review.

Statistical Data Analysis

Data was analysed using Resource Manager (RevMan) version 5.4.1 software (The Cochrane Collaboration, Copenhagen, Denmark). The statistical analysis of the systematic review involved evaluating the quality and risk of bias of the included studies. Retrospective studies were assessed using the Newcastle-Ottawa Quality Assessment Scale (NOS), where studies were rated on selection, comparability, and outcome. Randomised controlled trials (RCTs) were evaluated for bias using a risk-of-bias summary. Publication bias was assessed using a funnel plot.

Results

**Figure 1:**
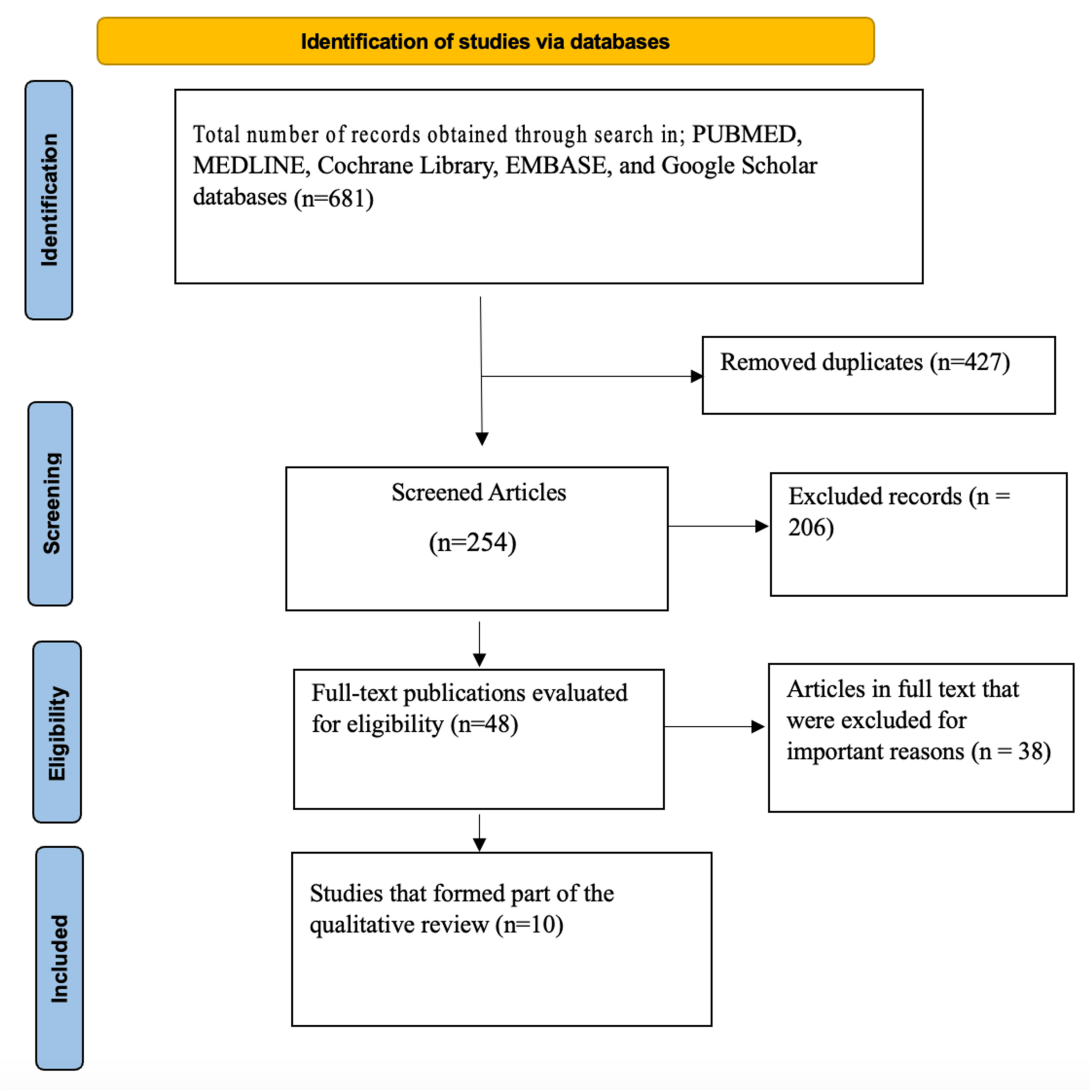
PRISMA flow diagram.

Figure 1 below presents a PRISMA flow diagram illustrating the selection process for studies included in this systematic review. The initial search across multiple databases yielded 681 articles: 312 from PubMed, 112 from Medline, 83 from the Cochrane Library, 47 from Embase, and 127 from Google Scholar. After removing 427 duplicate records, 254 unique studies proceeded to the initial screening phase. Of these, 206 studies were excluded based on predefined criteria. The remaining 48 studies underwent a thorough eligibility assessment, including 10 in the final systematic review.

Study Characteristics

The essential attributes of the studies included for review are detailed in the table below. All 12 publications used in this systematic review examined the role of NIV in managing AECOPD. All the studies included for review were conducted in various regions of the world and published in English.

**Table 1:** Characteristics of the included studies. AECOPD, acute exacerbation of chronic obstructive pulmonary disease; AHRF, acute hypercapnic respiratory failure; ASV, adaptive support ventilation; COPD, chronic obstructive pulmonary disease; HFNT, high-flow nasal therapy; IMV, invasive mechanical ventilation; LTH-NIV, long-term home non-invasive ventilation; NIV, non-invasive ventilation; NIPPV, non-invasive positive pressure ventilation; PaCO2, arterial partial pressure of carbon dioxide; PSV, pressure support ventilation; PtCO2, transcutaneous partial pressure of carbon dioxide; RCT, randomised controlled trial; QoL, quality of life.

Authors	Study type	Intervention	Sample size (N)	Outcome/results	Conclusion
Ralf et al., 2023 [10]	A retrospective analysis	Group 1: LTH-NIV treatment Group 2: non-NIV treatment	151	No significant difference in hospital readmission rate between cohort 1 and cohort 2, 1^st^ and 2^nd^ year survival rates were higher for cohort 1 compared to cohort 2(non-NIV-treated patients at 82% and 72%, respectively. Statistically significant mortality rate in non-NIV cohort.	LHT-NIV had significantly higher survival (lower mortality) rates than non-NIV interventions.
Stefan et al., 2015 [11]	Retrospective, multicenter cohort study	Cohort I: (patients enrolled on NIV) and Cohort II: patients who were first intubated with IMV	3,520	Hospital-based mortality was 7.4% and 16% for patients treated with NIV and IMV, respectively. NIV failure rate was 13.7% with an associated mortality rate of 22.5%. Prior NIV treatment was associated with a 41% lower risk of death.	NIV was associated with a lower mortality rate than IMV if prior interventions to manage AECOPD included NIV.
Ankjærgaard et al., 2016 [12]	Multi-center, open-label RCT	LT-NIV for intervention group and usual care for control group.	150	Patients were randomized to normal care or to continue with acute NIV in the long term, both groups being subject to a one-year follow-up period. Primary metric: duration to mortality or repeated AHRF; secondary endpoint: mortality within one year, number or readmissions, exacerbations, QoL, sleep quality, lung health.	Albeit conflicting evidence on LT-NIV treatment exists, it has been shown to reduce mortality and readmission on patients with prior need for acute ventilation support.
Elshof et al., 2023 [13]	Retrospective study	Adherence to guidelines for NIV in cases of respiratory acidosis among COPD patients	668	NIV was initiated for 76% of those admitted upon NIV indication. NIV was started for 65% of those whose health deteriorated after admission. Main reason for not starting NIV were – no signs of exacerbations and opted for comfort care.	NIV guidelines adherence is good. Physicians need more training to increase awareness to mitigate reluctance in initiating NIV on time.
Sehgal et al., 2019 [14]	RCT	NIV for patients with AECOPD in two modes: PSV and ASV	74	Primary outcome: NIV interventions failed (reinstitution of NIV within 48 hours of treatment termination, or mortality). Secondary outcome: mechanical ventilation was opted after NIV failed, both invasive and non-invasive. NIV failure rate was 28%. Adaptive support ventilation (ASV) reduced intubation rate by 9%. NIV applied using ASV resulted in similar success rate as pressure support ventilation (PSV).	The use of ASV in NIV yielded similar results in terms of success rate as PSV. Small sample size warrants the need for results to be confirmed in a larger clinical trial.
Sellares et al., 2017 [15]	RCT	120 COPD patients received either: 3 extra nights of NIV (n=61) or NIV discontinuation (n=59) after resolving an AHRF episode and tolerating unassisted breathing for 4 hours.	120	There was no significant difference observed in the relapse of AHRF between those on NIV and those discontinued, whose rates were 17% and for direct discontinuation and 13% for the NIV nocturnal cohort. Other metrics like long-term ventilation dependence, and length of hospital stay, were the same for the two cohorts.	Continuation of NIV post-recovery from AHRT does not prevent relapse in COPD patients. As long as patient can tolerate natural, unassisted breathing, NIV may be discontinued.
Faverio et al., 2019 [16]	Retrospective study	NIV weaning protocol: gradual interruption of 1 of 3 daily NIV sessions, starting from the morning session and finishing with the night session	51	Primary outcome: weaning completion – 39% of patients completed the NIV weaning protocol and were discharged; 39% failed to complete without NIV and were adopted to domiciliary ventilation under the chronic NIV cohort; 22% had NIV intolerance and weaning was terminated ex abrupto. Secondary outcome: AHRF relapse and frequency of delirium.	No relapse of AHRF reported among patients who successfully completed the weaning protocol. Interruption of an NIV session at a time is proposed as a novel weaning protocol.
Cortegiani et al., 2019 [17]	Multicenter, RCT	AHRF patients due to AECOPD assigned NIV or HFNT.	80	Primary outcome: reduction of PaCO2. Secondary outcome(s): arterial blood gases, vital parameters, respiratory rate, treatment intolerance and failure, need for endotracheal intubation, time spent under mechanical ventilation, ICU and hospital length of stay, and hospital mortality. Study established that HFNT was non-inferior to NIV in managing patients with acute AHRF patients due to AECOPD.	HFNT is relatively comfortable than NIV among AECOPD survivors. HFNT should be considered as an NIV alternative.
McKinstry et al., 2019 [18]	RCT	NIV and NHFT for treatment of patients with stable hypercapnic COPD.	24	Primary outcome: Change in PtCO_2_ at 60 minutes, adjusted for baseline. Secondary outcome: Proportion of participants with PtCO2 reduction ≥4 or ≥8 mm Hg, and participant ratings for ease of application, comfort, and fit of the therapies. No statistically significant difference in PtCO_2 _between the two groups.	NIV reduced PtCO_2 _better than NHFT among COPD patients. NHFT was preferred for ease of use, fit and comfort.
Shaheen et al., 2018 [19]	RCT	Oxygen and medical therapy for group I and NIV for group II	50	Group II registered a success rate of 76% and 20% for non-NIV group. Group I also registered more complications, including mortality rate.	NIV (NIPPV) has more benefits to AECOPD patients by reducing hospital stay, mortality and complications vis-à-vis standard therapy.

Assessment of Item Risk of Bias of the Retrospective Studies

The quality of the retrospective studies was evaluated using the Newcastle-Ottawa Quality Assessment Scale (NOS) (Table 2). A study was given to one-star rating for each numbered item in the selection and result categories. Comparability, however, was given up to a two-star rating. Two out of four retrospective studies were found to be of high quality with minimal risk of bias risk based on the assessment results [10,13]. However, 2/4 of the retrospective studies showed a high risk of bias in the follow-up period for an outcome to occur, and the adequacy of cohorts' follow. A study by Stefan et al. and Faverio et al. showed a high risk of bias regarding the follow-up period for outcomes and the adequacy of follow-up of cohorts’ outcomes [11,16].

**Table 2:** Assessment of quality of the included studies. For every numbered item in the selection and outcome categories, a study received up to one star (*). However, comparability received a rating of up to two stars. Based on the assessment results (**). For selection and outcome, (*) shows a low risk of bias. On the other hand, for comparability, (**) shows a low risk of bias, while (-) shows a high risk of bias.

Author	Selection				Comparability	Outcome		
	Representativeness of the exposed cohort	Selection of the non-exposed cohort	Ascertainment of exposure	Demonstration that outcome of interest was not present at start of study	Comparability of cohorts on the basis of the design or analysis	Assessment of outcome	Was follow-up long enough for outcomes to occur	Adequacy of follow-up of cohorts
Ralf et al., 2023 [10]	*	*	*	*	**	*	*	*
Stefan et al., 2015 [11]	*	*	*	*	*	*	-	-
Elshof et al., 2023 [13]	*	*	*	*	**	*	*	*
Faverio et al., 2019 [16]	*	*	*	*	*	*	-	-

Assessment of Item Risk of Bias of the RCT Studies

**Figure 2:**
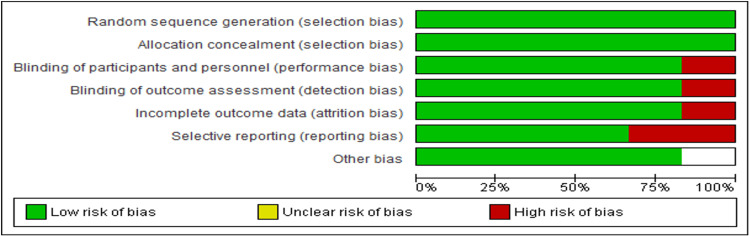
A summary of studies’ risk of bias of each item. In the risk of bias summary, which is based on the researcher's judgments of the risk of bias of the study items, red circles indicate high risk, green circles indicate low risk and white unclear risk of bias.

**Figure 3:**
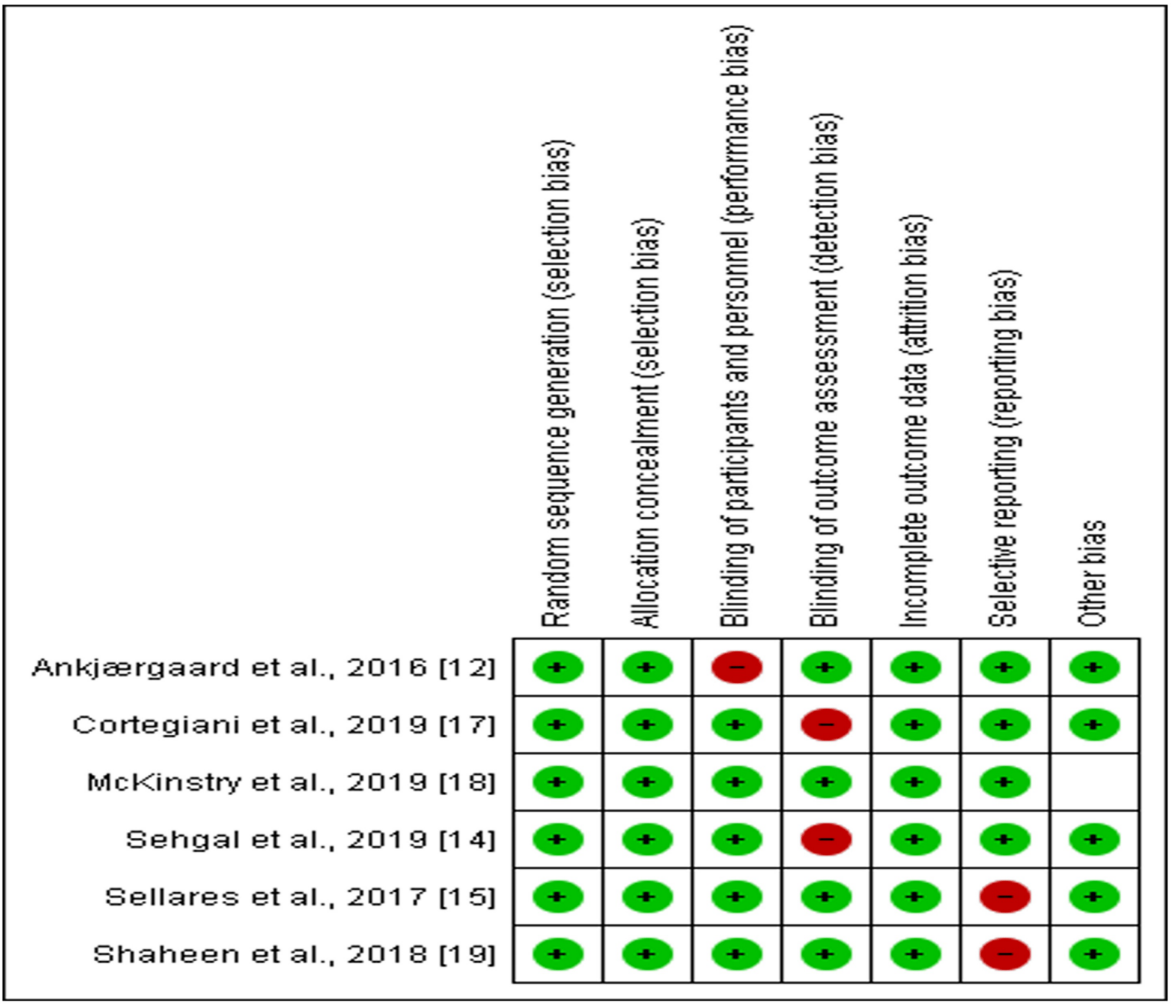
A summary of each studies’ risk of bias of each item.

Figure 2 and Figure 3 show that most of the six RCTs in this analysis were of good quality and had minimal bias risk. Using a random sequence generator minimised bias in selection, reporting, attrition, performance, and detection. However, 1/6 of the studies showed a high risk of performance bias [12], 1/6 of detection bias [14], and 1/6 of attrition bias [17]. On the other hand, 2/6 of the studies showed a high risk of reporting bias [15,19], while 1/6 showed an unclear risk of other biases [18].

Assessment of Publication Bias of the Included Studies

**Figure 4:**
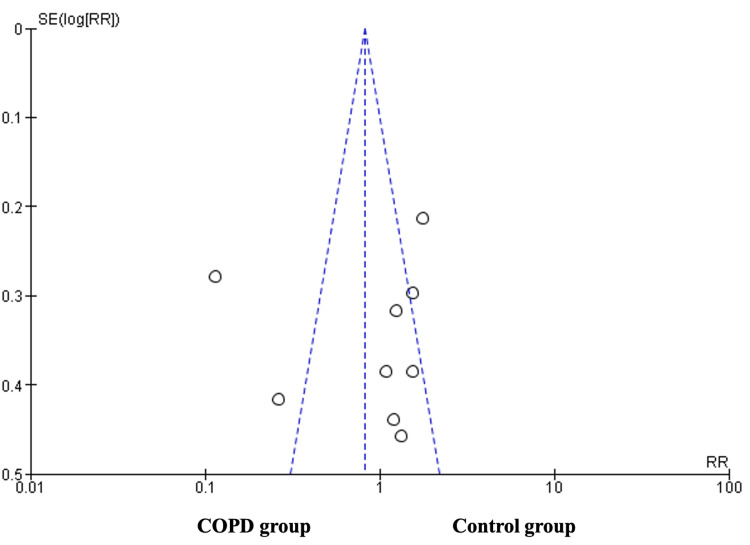
Funnel plot depicting publication bias. The standard error is denoted by SE and the relative risk by RR. X-axis, standard error; Y-axis, effect size. COPD, chronic obstructive pulmonary disease.

Figure 4 presents a symmetric distribution of effect sizes as a function of study precision in a funnel plot, with more studies appearing on the right. This shows a disproportionate number of studies on each side. This implies a possibility of publication bias in favour of the control group.

Discussion 

This systematic review aimed to evaluate the role of NIV in managing AECOPD. We evaluated the effectiveness, safety, and impact of NIV in managing exacerbated cases of COPD. Ten studies that fit our inclusion criteria for a thorough review were included. The studies provide substantial evidence supporting the use of NIV in managing AECOPD.

In line with previous studies, the studies under review point towards NIV significantly improving patient survival rates after acute exacerbated COPD. Ralf et al. demonstrated that long-term home NIV (LTH-NIV) led to higher survival rates than non-NIV treatments, with first and 2nd-year survival rates of 82% and 72%, respectively [10]. Stefan et al. established that NIV treatment for AECOPD was associated with lower mortality at the hospital than IMV [11]. Ankjærgaard et al. also proposed using LT-NIV as it was shown to reduce mortality and readmission in patients with a prior need for acute ventilation support [12].

Elshof et al. sought to examine levels of adherence and implementation of NIV guidelines in patients admitted with AHRF [13]. A high level of adherence to NIV guidelines was established, with 76% of patients admitted with respiratory acidosis being put under NIV. The study also found an even higher adherence rate for patients whose condition worsened upon admission and failure of IMV or other methods of managing AECOPD. The findings highlight the need for continuous training and awareness among healthcare providers to ensure optimal implementation of NIV guidelines.

NIV can be implemented in different forms, particularly pressure support ventilation (PSV) and adaptive support ventilation (ASV). However, these methods have different efficacy levels regarding reducing intubation rates; Sehgal et al. compared these modes and found that ASV reduced intubation rates by 9% compared to PSV [14]. Overall NIV effectiveness was nonetheless the same across the two modes of implementation. We can, therefore, conclude that the choice of which mode to use on a patient with respiratory acidosis or AECOPD depends on patient-specific factors and clinical settings.

Sellers et al. argued that relapse is still a possibility with or without weaning a patient off NIV [15] and that a patient can be discontinued from NIV as long as they can breathe unassisted after being stabilised. Conversely, Faverio et al. established a novel weaning protocol for AECOPD patients whose condition is stabilised through NIV [16]. Their study found that 39% of patients successfully weaned off NIV without relapse of AHRF during hospitalisation [16]. This underscores the importance of a structured NIV weaning protocol to safely transition AECOPD patients back to unassisted breathing, with minimal risk of relapse and faster hospital discharge.

HFNT has stood its ground and proved to be a viable, non-inferior alternative to NIV. Cortegiani et al. established that as far as comfort is concerned, HFNT should be considered an NIV alternative [17]. McKinstry et al. established that while NHFT was preferred for ease of use, fit and comfort, NIV reduced PtCO2 better than NHFT among COPD patients [18]. Both methods yielded similar efficacy in reducing PaCO2. HFNT could, therefore, be considered for patients who may not tolerate NIV well, providing flexibility in treatment options. 

Studies comparing NIV and IMV highlighted the superiority of NIV in treating AECOPD. For instance, Stefan et al. established that NIV was associated with lower mortality rates (7.4% for NIV-treated patients versus 16% for IMV-treated patients), alongside other benefits like reduced need for intubation, and fewer complications [11]. Failure of NIV intervention and delayed treatment led to increased intubation rates, which were associated with higher mortality and more post-intubation complications. Similar to Stefan et al. findings, Shaheen et al. concluded that patients stood to gain more from NIPPV through reduced mortality rates, early discharge from the hospital, and fewer complications vis-à-vis IMV and other standard therapy interventions [11,19]. This evidence underscores the need for awareness and timely intervention using NIV methods in managing AECOPD.

## Conclusions

We conclude that, in light of the empirical literature evidence reviewed herein, non-invasive ventilation (NIV) is a highly effective and safe treatment for managing acute exacerbation of chronic obstructive pulmonary disease (COPD). The studies reviewed demonstrated that NIV significantly improves patient survival rates; long-term home (LTH)-NIV showed higher survival rates after exacerbation compared to invasive mechanical ventilation (IMV) treatment. NIV was consistently associated with reduced mortality, shorter hospital stays, lower intubation rates, and fewer complications compared to IMV. Adherence to guidelines and structured weaning protocols further enhance the effectiveness of NIV. High-flow nasal therapy presents a viable alternative for patients with stable or exacerbated cases of COPD who may not tolerate NIV. However, the NIV protocol under review is novel and requires testing on a larger scale. Further research on standardising treatment and weaning protocols to fortify existing NIV guidelines and explore patient-specific factors to optimise NIV application in clinical practice.
